# Detection of canonical A-to-G editing events at 3′ UTRs and microRNA target sites in human lungs using next-generation sequencing

**DOI:** 10.18632/oncotarget.6132

**Published:** 2015-10-15

**Authors:** Ramani Soundararajan, Timothy M. Stearns, Anthony J. Griswold, Arpit Mehta, Alexander Czachor, Jutaro Fukumoto, Richard F. Lockey, Benjamin L. King, Narasaiah Kolliputi

**Affiliations:** ^1^ Division of Allergy and Immunology, Department of Internal Medicine, Morsani College of Medicine, University of South Florida, Tampa, FL, USA; ^2^ MDI Biological Laboratory, Salisbury Cove, ME, USA; ^3^ Center for Genetic Epidemiology and Statistical Genetics, John P. Hussman Institute for Human Genomics, University of Miami, Miller School of Medicine, Miami, FL, USA

**Keywords:** RNA editing, RNA-seq, exome, 3′UTRs, microRNAs

## Abstract

RNA editing is a post-transcriptional modification of RNA. The majority of these changes result from adenosine deaminase acting on RNA (ADARs) catalyzing the conversion of adenosine residues to inosine in double-stranded RNAs (dsRNAs). Massively parallel sequencing has enabled the identification of RNA editing sites in human transcriptomes. In this study, we sequenced DNA and RNA from human lungs and identified RNA editing sites with high confidence via a computational pipeline utilizing stringent analysis thresholds. We identified a total of 3,447 editing sites that overlapped in three human lung samples, and with 50% of these sites having canonical A-to-G base changes. Approximately 27% of the edited sites overlapped with Alu repeats, and showed A-to-G clustering (>3 clusters in 100 bp). The majority of edited sites mapped to either 3′ untranslated regions (UTRs) or introns close to splice sites; whereas, only few sites were in exons resulting in non-synonymous amino acid changes. Interestingly, we identified 652 A-to-G editing events in the 3′ UTR of 205 target genes that mapped to 932 potential miRNA target binding sites. Several of these miRNA edited sites were validated *in silico*. Additionally, we validated several A-to-G edited sites by Sanger sequencing. Altogether, our study suggests a role for RNA editing in miRNA-mediated gene regulation and splicing in human lungs. In this study, we have generated a RNA editome of human lung tissue that can be compared with other RNA editomes across different lung tissues to delineate a role for RNA editing in normal and diseased states.

## INTRODUCTION

In recent years, the focus of molecular biology has been radically shifted from the “central dogma” (DNA to RNA to protein) to encompass the role of modifications of RNA nucleotides via co- or post-transcription mechanisms termed “RNA editing”. RNA editing involves alteration in the sequence of RNA that is derived from DNA. The diverse type of RNA editing events leads to different functional consequences [1]. RNA editing was first discovered in the trypanosome in an mRNA encoded by the kinetoplastid mitochondria [2]. This phenomenon was later discovered in the nuclear-encoded mRNAs in the mammals [3]. There are two types of canonical RNA editing found in the nuclear mRNAs. One involves the deamination of Cytidine (C) to Uridine (U) that is relatively less common and catalyzed by members of cytidine deaminase (AID/APOEBC) family of proteins [4, 5]. The second type and the most prevalent RNA editing event in mammals involves site-specific deamination of Adenosine (A) to Inosine (I) in dsRNA that is catalyzed by adenosine deaminase acting on RNA (ADAR) family of proteins [6]. The consequence of this base change is the recognition of inosine as guanosine by the translational machinery leading to A-to-G editing [6]. In addition, as inosine base-pairs with cytidine, the structure of RNA can be altered by ADARs by changing AU base pairing to IU mis-match [1]. Mammalian ADAR proteins (ADAR1 and ADAR2) are ubiquitously expressed, whereas ADAR3 is specifically expressed in the brain [1]. All ten other types of possible editing events are classified as non-canonical and are not associated with any known enzymatic process. Non-canonical events may be an artifact of high throughput sequencing, and recent studies show a lack of their validation via Sanger sequencing [7].

RNA editing can occur in either coding or non-coding regions of dsRNA. The site-specific deamination of adenosine residues in pre-mRNAs can alter the individual codons resulting in protein diversity. Moreover, ADARs can edit multiple sites on the same mRNA species thereby further diversifying the functional outcomes [8]. RNA editing in coding regions may result in recoding and functional diversification of proteins as seen with neurotransmitters and ion channels in brain [9-11]. Additionally, RNA editing in introns near a splice site may affect splicing. Non-coding regions of genes that are edited include the UTRs [6]. The majority of RNA editing observed in non-coding regions involve repetitive elements, such as a long interspersed nuclear element (LINE) or small interspersed nuclear element (SINE) [12]. However, the biological significance of repetitive element editing is unknown. In the UTRs, editing by ADARs can either abolish existing microRNA (miRNA) binding sites or generate new miRNA target sites owing to seed sequence differences in miRNA, thereby suppressing a different set of target genes [13]. RNA editing in ncRNAs may alter RNA structure or stability, leading to biological consequences. A-to-I editing of pri- or pre-miRNA by ADAR1 and ADAR2 inhibits their processing to mature miRNAs [13]. This information suggests a potential regulatory role and functional consequence for RNA editing.

Dysregulation of RNA editing has been linked to several neurodegenerative diseases such as epilepsy, amytrophic lateral sclerosis (ALS), depression, brain ischemia and tumor [8, 14], and human cancers [14-18]. Decreased A-to-I editing has been observed in tumors of brain, lung, kidney and testis [16]. The highest levels of ADAR1 transcript are observed in brain and lungs relative to other tissues [19]. However, unlike brain, the RNA editome of normal lung has not been well studied. In a previous study, ADAR1 was shown to be upregulated in a microvascular lung injury mouse model of inflammation suggesting a role of RNA editing in pathogenesis of acute lung injury [20]. Therefore, we sought to understand the editome of normal human lungs to later address the potential role of RNA editing in lung pathogenesis.

With the advent of high-throughput sequencing (HTS) technology and developmental of computational tools, it has been possible to identify RNA editing sites in human and mouse transcriptomes [7, 12, 16, 21-23]. RNA-seq data alone or RNA-seq combined with DNA-seq data can be used to identify RNA DNA difference or RNA editing sites by comparing RNA sequences with the annotated human reference genomic sequences [24-26]. There are many challenges that are faced by researchers in the field of RNA editing to call true variants versus minimizing the identification of false positives in HTS data [27]. The identification of false positives may be attributed to sequencing or mapping errors. Conversion of RNA to cDNA can result in mutation and be a source of false positive editing sites. Others include errors in reference genome, paralogous genes, alignment errors at splice junction sites and introns, identification of SNPs as editing sites and hard to map heavily edited repetitive Alu elements or genes that are edited but expressed at low levels [27]. There are several strategies that can be applied to overcome identification of false positives in RNA editing analysis by accurately mapping reads to introns and splice junctions, applying stringent computational pipelines, trimming first six base pairs of reads, removing all known SNPs from the datasets, using RNA and DNA sequences from the same sample and validating the editing sites by biochemical or molecular biology techniques [27].

In the present study, we investigated RNA editing in three normal human lungs using high-throughput (exome) DNA- and RNA-sequencing data from the same sample and computational pipeline. We mapped reads to reference genome and that included all splice junctions and introns for unique mapping of reads. We used a stringent computational pipeline to identify RNA editing sites in three normal lung samples with REDITools software package [28] (http://150.145.82.212/ernesto/reditools/doc/) using the criteria: minimum depth of coverage 10X in DNA and RNA, no variant alleles in the DNA sequencing, at least 2 alternate alleles and at least 10% alternate alleles in RNA sequencing. These criteria were based on information available from previous publication [23]. These criteria led to extraction of RNA editing sites that overlapped across all 3 samples with the same substitution. We also removed all possible SNPs from the data using the dbSNP database.

This study demonstrates that RNA editing is widespread in normal human lungs, and the majority of events are canonical A-to-G editing that map to introns and 3′ UTRs of target genes. We observed few non-synonymous amino acid changes in target genes as very few edited sites were found in exons or coding sequence (CDS) of target genes. Many of the hyper-edited sites were found in A-to-G clusters. Several of these editing sites were validated by Sanger sequencing. Interestingly, we identified editing within the miRNA binding sites in 3′UTR of candidate genes for normal lung samples and several miRNA edited sites were validated by *in silico* method. Altogether, for the first time, we have generated an editome of normal human lungs using high-throughput sequencing technology and computational tools. This database would serve as an important platform to discern a role of RNA editing in normal lung biology and its dysregulation in lung disease.

## RESULTS AND DISCUSSION

### RNA-sequencing and whole exome analysis of lung tissue

We sequenced DNA (exome plus UTRs) and RNA from three normal human lung samples using the Hiseq2500 platform (Illumina). For DNA sequencing, we generated an average of 110 million reads per sample of which ∼98% aligned uniquely to the human reference genome (hg19). 79% of reads aligned to the targeted exome giving an exome coverage of 69X. 92% of targeted bases were covered at 10X coverage and 81% at 20X (data not shown). For RNA sequencing, we generated an average of 130 million reads per sample of which ∼88% mapped to the human reference genome (hg19) with only 5% of the reads mapping to multiple loci (data not shown). For each lung sample, we identified both the canonical and non-canonical editing sites by comparing their own DNA and RNA sequences using REDItools as previously described [28, 29]. Further filtering was done using criteria as previously described [23]: 10X minimum depth of coverage in both DNA and RNA sequences, no variant alleles in DNA sequencing, and at least 2 alleles and 10% alternate alleles in RNA sequencing (Figure S1). We extracted sites that overlapped across all three normal lung samples with the same base substitutions. We retained Alu sequences in our analysis as they represent potential sites of ADAR-mediated editing [22]. Previously, several groups have identified RNA editing event in human tissues using only RNA-seq data alone without the DNA sequence information and developed various computational pipeline to minimize the detection of false positive editing sites in their datasets [12, 16, 21, 22, 26, 30, 31]. As of now, there is no fool-proof single computational tool that is most appropriate in detecting RNA editing sites with high confidence. We acknowledge that comparison between different platforms is difficult to make an argument as to which is the best computational tool for editing analysis. However, RediTools is widely used tool for studying RNA editing either using RNA-seq data alone or both RNA-seq and DNA-seq data. The software has various filters to minimize biases resulting from sequencing errors, mapping errors, and SNPs [28].

**Figure 1:**
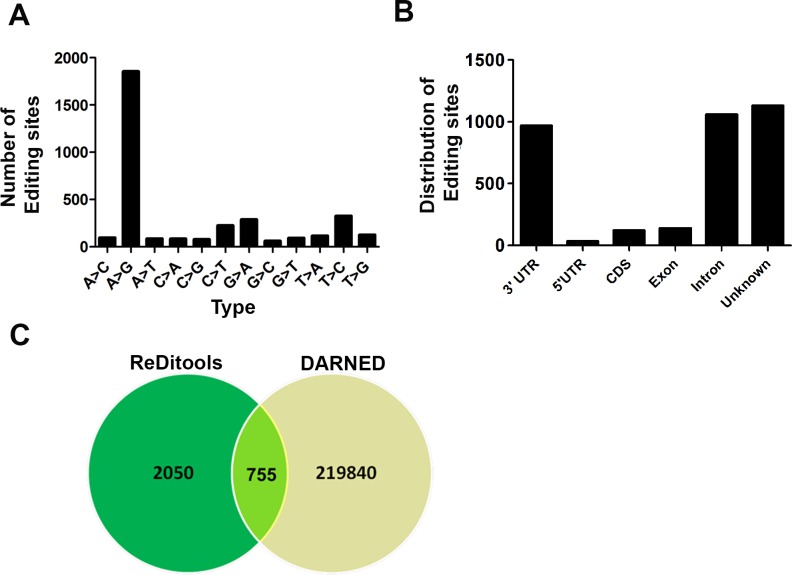
Identification of RNA editing sites in normal lung samples **A.** Both canonical (A-to-G) and all other types of non-canonical events that overlapped between all three normal lung samples are shown. **B.** Genomic distribution of all overlapping RNA editing types in the three normal human lung samples. **C.** Venn diagram showing the overlap between edited sites identified in our analysis and DARNED.

At 10X coverage, we identified 5,538 edited sites that were present in all the three lung samples, and of these 2,805 edited sites were A-to-G, 344 were C-to-T and 2,389 were all other types of base substitutions. For more confident filtering of edited sites, we used 20X coverage and 20% alternate RNA allele frequency and identified 3,447 sites that were present in the three human lung samples. These included both canonical editing sites (1,856 A-to-G and 226 C-to-T) and all other types (1,365) of non-canonical editing sites (Figure 1A). We report 50% (1,856/3,447) of canonical A-to-G editing sites in our lung dataset, which correlate well with other published reports that used high-throughput human sequencing data from various tissues or cells [21-23]. In addition to the overlapping editing sites, we also found 1,480, 1,594 and 1,853 unique A-to-G editing events in each of the three human lung samples, respectively; this implies that within normal human lung samples, there is some degree of heterogeneity with respect to the editing events. Previously, the ENCODE project utilized RNA-seq data only to identify 50-85% of RNA variants (A-to-G substitutions) in 14 human cell lines. An important finding from this project is that although the list of genes with the edited sites overlapped between different cell lines, the individual sites that were edited varied, implying tissue-specific editing events. In the ENCODE project, a total of 1,322 RNA variants were identified in normal human lung fibroblasts (NHLF) and 80% of these were A-to-G base changes. We identified 1,856 A-to-G base changes in normal human lung tissues using both RNA and DNA sequencing data after filtering for SNPs. These datasets suggest that there could be a difference in cell-type-specific editing in lungs, although this cannot be discerned from currently available public data. There are several steps inherent to identification of RNA editing sites by high throughput sequencing that can result in false positive results. These include molecular events, such as the introduction of variants by RNA to cDNA conversion, and bioinformatic events such as misalignment, mapping errors in paralogous genes, and rare reference variants that appear as edited events. We have adopted several steps, suggested by Bass *et al*., 2012, to reduce false positives. First, we have sequenced RNA and DNA from the same tissues removing the possibility that rare DNA variants are interpreted as editing events. Secondly, we have adopted a strict two-pass alignment strategy adopted as the GATK best practice for calling sequencing variants from RNA (https://www.broadinstitute.org/gatk/guide/article?id=3891). Finally, we have utilized a strict heuristic filter to reduce false positive including coverage of at least 20X, 20% alternate allele frequency, and absence of the site in the DNA sequencing. Identification of a vast majority of canonical A>G editing sites suggests that overall false positive rates are low as reported in a recent paper [12]. While we have not directly calculated the false discovery rate in our data, we believe it to be as good as or better than previously published estimates.

The profiling of a RNA editome of a lymphoblastoid cell line, which was derived from a single human individual using RNA-seq data, led to the identification of 22,688 editing events. Majority (21,099) were A-to-G editing events, and the rest were all other types of non-canonical editing events [22]. In a recent study [23], RNA-DNA differences identified in a B-lymphoblast cell line derived from two humans subjects revealed approximately 10,000 overlapping A-to-G editing events. Using a similar approach, we identified 1,856 A-to-G overlapping editing sites in normal human lung tissues. This disparity in the number of editing sites may be due to the editing levels in cultured B-lymphocytes relative to lung tissue, and heterogeneity of lung tissues compared to the homogenous B-lymphocyte culture. In another large-scale deep-sequencing study [31], 5,965 out of a total of 9,636 editing sites were identified as putative A-to-G events. In our study, we identified 1,856 A-to-G editing events in normal human lung tissue. The variation in results among published studies may be due to a number of factors including the use of differing analysis pipelines and filters, study designs, sequencing depth or coverage, distribution of editing sites, and extent of transcriptome analyzed. Re-analysis of a controversial published data [32] by another group lead the authors to conclude that large number of sites called positive for editing were not validated by their analysis [33]. From these studies we can conclude that there is a need for the development of a common analysis with stringent filtering criteria to facilitate comparisons across different editome analyses and avoid identification of false positive editing sites. In addition, there is a need to validate the editing sites by molecular biology and biochemistry techniques *in vitro* instead of relying completely on computational analysis alone. Similar to human studies, RNA-editing analysis in different tissues of mice identified 242 A-to-G editing events. All other non-canonical editing sites (683) were shown to be an artifact of the sequencing reaction [7]. Taken together these studies support the view that a large proportion of editing events in mammals are canonical A-to-G base changes.

### Genomic distribution of canonical and non-canonical editing sites in lungs

Further analysis of all overlapping canonical (1,856 A-to-G and 226 C-to-T) and non-canonical editing (1,365) events in lung tissue at 20X coverage and 20% alternate alleles revealed enrichment in the 3′ UTR and introns of genes consistent with previous findings. Conversely, the coding sequences represented only a small fraction of edited sites in the target genes that is consistent with previous reports [22, 23]. In our datasets around 1,050 sites ( 30%) mapped to introns, 968 sites (28%) to 3′ UTR, 138 sites (4.0%) to exons, 120 sites (3.5%) to coding sequences (CDS), and 1,130 edited sites (32.78%) could not be assigned to any gene by annotation and represent unknown editing events (Figure 1B). In a recent study, RNA editing sites in brain, thyroid, lung, heart and skeletal tissue (tissue specific editing, TSE) were found to be enriched in 3′ UTR of genes [30]. This is in agreement with our analysis of lung editome showing enrichment of editing sites in 3′ UTR of genes. In our dataset, only 8 out of 200 (4%) overlapping editing sites in exons resulted in a non-synonymous amino acid change. The A-to-G editing in *Dcp2*, *Pms2*, *Senp3*, and *Znf551* resulted in non-synonomous amino acid change.

In the ENCODE project, the A-to-G editing sites in the lymphoblastoid cell line similarly mapped to introns (51%) and 3′UTRs (39%) as observed in our dataset; whereas in the non A-to-G editing sites, there was a 82% enrichment near splicing boundaries [21]. Similar to Peng et al. (2012), we found that the sites in the transcripts were edited to varying levels in both coding and non-coding regions. We observed that genes with single or multiple edited sites mostly mapped to either 3′ UTR or introns relative to exons or CDS. We also found that at 10X coverage, 1,517 (27%) sites out of 5,538 overlapped with Alu-rich regions of genes. Consistent with previous published observations [22, 23], the A-to-G base changes at multiple sites were enriched in sequences that significantly overlap with Alu and LINE or SINE elements. An interesting feature of the edited sites in the target genes in our dataset was the A-to-G site clusters (30% of sites patterned in >3 clusters in 100 bp). This is in concordance with previous reports [22, 34] but substantially differs from what is reported (85%) in the DAtabase of RNA Editing (DARNED) [35]. DARNED database is a database of RNA editing in humans. It provides centralized access to all publications related to RNA editing. The latest release contains 333,215 edited sites, of which 221,595 are A-to-G edited sites. The database contains information on the tissue, organ or cell wherein the editing has been observed, the gene that is edited, the co-ordinates and information on the SNPs. We further compared the sites that were edited in our dataset with editing sites in DARNED (http://darned.ucc.ie/download) [35]. We found 755 (26.92%) A-to-G edited sites out of a total of 2,805 from our (REDItools-generated) dataset in DARNED (Figure 1C). Therefore, 2,050 (73.08%) editing sites are uniquely identified from the analysis of our lung data. DARNED contains a total of 333,215 editing sites, 220,604 (66.20%) of those sites are unique A-to-G type. Hence, our dataset identified 0.34% (755/220,604) of the unique A-to-G DARNED sites. The low percentage of edited sites that overlapped with DARNED is significant considering that DARNED is not enriched for lung-specific genes. This is in concordance with previous publications wherein a low percentage of edited sites were shown to overlap with DARNED [22, 34]. Taken together, this information suggests that some edited sites may be present in all tissues but the majority of ADAR-mediated editing may be tissue or cell-specific. Moreover, ADAR may target the same transcript at different positions in a tissue or cell-dependent manner. As we were interested in only ADAR-mediated canonical editing, we further studied the ADAR-specific targets in the lung.

### Characterization of ADAR-specific target sites in lungs and pathway enrichment of edited genes

For the functional characterization of target sites, we focused on A-to-G canonical editing events in lung tissue that are likely to be mediated by ADARs. We mapped the edited sites that overlapped in all three normal lung samples to identify 513 unique genes with at least one edited sites at 20X coverage. We have listed all the genes, the chromosomal region showing the edited sites, type of editing and genomic location in Table S1. It is evident from Table S1 that 24 (2.61%) of the 513 genes are hyper-edited - having 10 or more editing sites; whereas the rest of the genes have < 10 edited sites. To discern the functional role of ADAR1 or ADAR2, the future studies will address the effect of ADAR1/ADAR2 knockdown on editing in lung fibroblasts derived from normal human lungs.

### Identification of RNA edited sites by REDItools analysis

**Figure 2:**
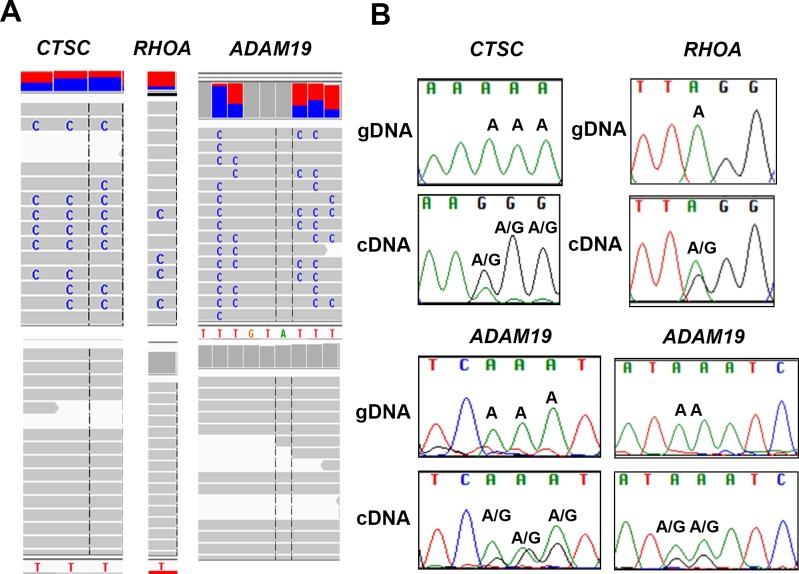
Validation of A-to-G editing by Sanger sequencing **A.** Integrative Genome view of *Ctsc*, *RhoA* and *Adam19* is shown. The RNA- and DNA-seq traces are shown in top and bottom panel, respectively. **B.** Validation of editing sites in *Ctsc*, *RhoA* and *Adam19* is shown. For *Ctsc*, three editing sites were validated (chr11:88,055,689, 88,055,690, 88,055,691). For *RhoA*, we validated one site (chr3:49,397,323). For *Adam19*, we validated five sites chr5: (156,905,567, 156,905,566, 156,905, 565 in the left panel and 156,905,561, and 156,905,560) in the right panel. The sites validated by Sanger are shown and the nucleotide changes are labelled. Samples were sequenced using both forward and reverse M13 primers. We show few representative images of each.

Editing sites in genes were analyzed using Integrative Genomics Viewer (IGV). The edited sites in *Ctsc*, *RhoA* and *Adam19* were visualized by IGV (Figure 2A). IGV images reveal editing sites (C) only in the RNA-seq trace (upper panel) and not in the corresponding genomic trace (lower panel). These represent putative editing sites (in negative strand) (Figure 2A).

### Validation of edited sites by Sanger sequencing

To validate our findings *in vitro*, we randomly selected (A-to-G) canonical editing sites comprising both hyper- and single-edited sites in the 3′ UTR regions of genes for Sanger sequencing. For the hyper- or single-edited sites, we PCR amplified 400bp region flanking the edited sites using specific primers tagged with M13 sequence to amplify both genomic DNA and cDNA. Sanger sequencing was performed for each PCR-amplified product (Genomic and cDNA) using both forward and reverse M13 primers for all three lung samples resulting in a total of twelve sequences per transcript. These sequence files (AB-1) were aligned and further analyzed using Sequencher 5.1 software (Gene Code Corporation). We validated several canonical editing sites in all three lung samples *in vitro* by Sanger sequencing. We show representative images for *Ctsc*, *RhoA* and *Adam19,* respectively (Figure 2B). The genomic and cDNA trace of the edited sites for each gene is shown (Figure 2B). For *Ctsc*, we show three edited sites (chr11:88,055,689 to 88,055,691). For *RhoA*, we validated A-to-G editing at a single site (chr3:49,397,323). Similarly, for *Adam19*, we confirmed A-to-G editing at five different sites (chr5:156,905,556 - 156,905, 568). For sites that were validated, an “A” (*un-edited reference peak*) in the genomic trace and two peaks in cDNA trace, namely an “A” (*un-edited reference peak*) and a “G” (*edited peak*) were observed in the positive strand (Figure 2B). Taken together, these results indicate true editing events occurring in normal human lung tissue, and, most interestingly, the canonical edited sites are located in the non-coding regions of the genes.

For pathway analysis, we further filtered 513 edited genes by selecting all genes in the overlap with at least one 3′ UTR editing site and this resulted in 336 genes in the dataset. We performed gene set enrichment using MetaCore and BinGo. Interestingly, lung-specific editing sites were found in genes related to Apoptosis and cell survival, cytoskeleton remodeling, ER stress response pathway, Granzyme B signaling, TGF-beta, Wnt and Erk signaling (Table S2). This is contrast to a study wherein they found lung-specific editing sites enriched in genes related to signal peptide processing and response to viral or inflammatory stimuli [30]. Gene ontology (GO) revealed enrichment of several metabolic processes (Table S3). The small number of genes that are enriched in these pathways suggests that the edited events may not be pathway specific but represent similar genes that are edited in the overlapping dataset and may thus represent baseline editing in normal human lungs.

### Role of RNA editing in miRNA-mediated regulation of candidate genes

**Figure 3:**
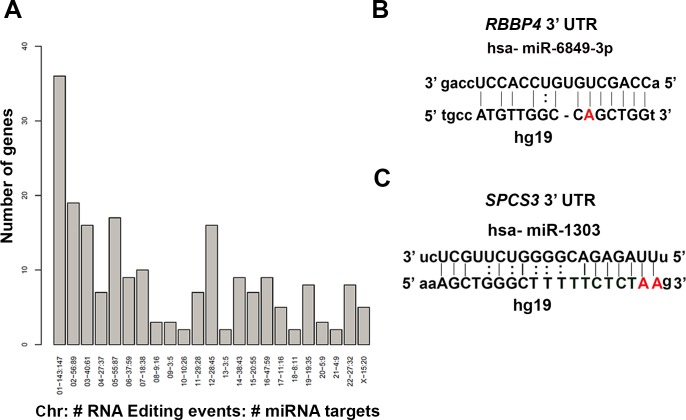
Identification of RNA editing in miRNA target binding sites in the 3′ UTR of genes **A.** Genes containing A-to-G edited miRNA targets for all chromosomes are shown. On the x-axis, the chromosome number, total number of editing events, and total number of miRNA targets associated with it are listed. On the y-axis, the number of genes associated with editing events in the miRNA binding sites is depicted. **B.**-**C.** Validation of editing in potential miRNA binding sites by *in silico* analysis for *Rbbp4* **B.** and *Spcs3* **C.** The images show miRNA target sites and the complementary hg19 sequence. The edited site in the seed sequence of the mature miRNA is highlighted in bold. The hsa-miR-6849-3p binding site was located in *Rbbp4* 3′ UTR at chr1:33,149,343-586. The bold position is an A-to-G RNA-editing event located at chr1:33,149,443 in *Rbbp4* (B). Similarly the hsa-miR-1303 binding site is located in *Spcs3* 3′ UTR at chr4:177,252,226-427. The bold positions are two A-to-G edited sites located at chr4:177,252,326 and 327 (C)..

Given that the majority of edited sites identified in the lung samples were in the 3′ UTR of the candidate genes, we were interested in determining if these sites were located within miRNA target sites. The potential role of RNA editing in miRNA-mediated gene silencing has been previously described [13, 36]. We identified potential human miRNA target sites (energy < −20 and score > 155) associated with Ensembl (GRCh37) transcripts using miRanda [37]. Using Ensembl BioMart (GRCh37), each transcript with only one validated 3′ UTR start site had its position reported. For each of those transcripts, the BioMart-reported 3′ UTR start position was added to the miRanda-reported RNA start and end coordinates to obtain the miRNA target locations. We then parsed the generated miRNA target locations to determine if the previously identified RNA edited sites existed within those boundaries. As we were interested in only A-to-G canonical editing sites, we filtered for those events and generated a list of genes containing A-to-G editing in miRNA targets (Figure 3A). For all chromosomes analyzed, we found a total of 652 editing events in 933 potential miRNA target binding sites that mapped to 205 candidate genes (Figure 3A). From Figure 3A, it is evident that miRNA sites in various chromosomes are edited differentially. The most logical explanation is that these target genes are differentially regulated in the three normal human lung samples. The data on the miRNA binding sites in the target genes that are edited, and the chromosomal location are shown in Table S4. As shown in Table S4, multiple miRNA binding sites are edited for majority of genes relative to single edited site. Considering that we identified 513 candidate genes that were edited in all three lung samples, 39.96 % (205/513) of candidate genes have miRNA binding sites edited in their 3′ UTRs. In a previous study [22], 20.89% of the edited sites that resided in the 3′ UTR were reported to alter the miRNA target sites. In another study [38], RNA editing was identified in 6% of human miRNA and was proposed to affect the miRNA processing possibly resulting in altered target recognition and increased miRNA diversity. Nevertheless, the functional consequence or biological significance of altered miRNA target sites (single or hyperedited) on gene expression remains to be addressed. From the literature, it is apparent that in human transcriptome, majority of sites (>85%) are hyper-edited in Alu repeats. Alu elements have been shown to modulate gene expression at post-transcriptional level. These repetitive elements easily form double stranded structures and provide ideal substrate that is amenable to editing by ADAR enzymes. Several groups have used HTS and computational pipeline with stringent filtering to identify A-to-G editing in Alu repeats [24-26]. Site-specific editing in introns may affect splicing or in codons, alter the amino acids composition and thereby increase protein diversity. In the 3′ UTR, site-specific RNA editing can alter miRNA binding sites thus affecting gene expression [6].

We randomly selected few miRNA target sites in genes and validated the alteration in the miRNA binding sites (within 2^nd^ to 8^th^ nucleotide of the seed region of mature miRNA) by an *in silico* approach using miRanda. As an example, we show the A-to-G editing in the miRNA binding sites for two genes *Rbbp4* and *Spcs3* (Figure 3B-3C). For *Rbbp4*, hsa-miR-6849-3p binding site is located in chr1 between the positions 33,149,343-586 in the 3′ UTR of the gene. The miRNA sequence is shown at the top and the complementary hg19 region is shown below. The A-to-G editing site mapped to chr1:33,149,443 (7^th^ nucleotide of the seed) (Figure 3B). Similarly, for *Spcs3*, we identified hsa-miR-1303 binding site in chr4:177,252,226-427 in the 3′ UTR of the gene (Figure 3C). The A-to-G edited sites mapped to chr4:177,252,326 (1^st^) and 327 (2^nd^) within the seed sequence (Figure 3C).

In summary, we have generated an editome of normal lung tissue that defines the RNA variants overlapping in three samples using (exome) DNA- and RNA-sequencing data and REDItools RNA editing analysis. In future studies, we will adapt this methodology to interrogate large datasets of lung tissues to generate a RNA editome of normal and diseased samples. Profiling the RNA editome will assist in understanding the role of RNA editing in both normal and diseased states, and will be a novel approach to understanding the molecular basis of lung pathogenesis.

## MATERIALS AND METHODS

### Ethics statement

This study was reviewed and approved by the Institutional Review Board (IRB) of the University of South Florida.

### Human samples

De-identified normal human lung tissues (N=3) collected from peripheral regions of lungs from deceased individuals, 40-60 yrs of age ( 2 males and 1 female) were obtained from National Disease Research Interchange (NDRI), Philadelphia, PA. These “normal human lung” tissues refer to the samples obtained from non-diseased individuals.

### Sample preparation

Genomic DNA was extracted from three normal human lung samples using DNeasy kit as per manufacturer's instructions (Qiagen). Total RNA was extracted from three normal human lung samples using Trizol reagent (Life Technologies) combined with RNeasy kit as per manufacturer's instructions (Qiagen). To prevent genomic DNA contamination of RNA, we treated RNA samples to on column DNase digestion according to manufacturer's instructions (Qiagen). Quality control for both genomic DNA and RNA was carried out using Agilent 2100 Bio-analyzer at the Center for Genomic Technology (CGT), John P. Hussman Institute for Human Genomics at the University of Miami Miller School of Medicine.

### Massively parallel sequencing

Massively parallel sequencing (whole exome sequencing and RNA sequencing) was performed at the CGT. DNA-seq libraries for three normal human lung samples were prepared with Agilent Xt reagents and captured with the Agilent SureSelect 50MB + UTR exome V5 sample kit. RNA-seq libraries were prepared for three normal human lung samples using the Epicenter Ribozero ScriptSeq-v2 RNA prep kit (Illumina). All samples were sequenced 3 per lane with paired end 100bp reads on a HiSeq2500 (Illumina).

### Analysis for identifying RNA editing sites

### DNA alignment and variant calling

DNA reads were processed according to Genome Analysis Toolkit (GATK) best practices. DNA reads were aligned to the human hg19 (GRCh37) genome with bwa and exome enrichment statistics calculated with PICARD. DNA variants were called with the GATK Unified Genotyper for each individual exome sample. Variants were filtered for sites at a minimum depth of 8X and PL score > 100 for alternate alleles. We used ANNOVAR for annotation and included annotation from RefGene, dbSNP, frequency in the Exome Variant Server (EVS), frequency in 1000 Genome project, and frequency in the Exome Aggregation Consortium (ExAC).

### RNA Alignment

RNA reads were processed for variant calling according to the GATK best practices including quality and adapter trimming with TrimGalore, alignment in a 2-pass alignment with the STAR aligner to the human genome hg9/GRCh37, and base quality recalibration.

### Identification of RNA editing sites with REDItool

We used the REDItools software package to create tables of every potential RNA editing position (http://150.145.82.212/ernesto/reditools/doc/) [28]. We required: minimum depth of coverage 10X in DNA and RNA, no variant alleles in the DNA sequencing, at least 2 alternate alleles and at least 10% alternate alleles in RNA sequencing based on previous publication [23]. These criteria led to extraction of RNA editing sites that overlapped across all 3 samples with the same substitution. Annotation of each editing sites in the selected files was achieved by using the Human RefSeq database. Confidence filtering for edited sites was achieved by using stringent conditions such as 20% alternate RNA allele frequency and 20x RNA coverage. Feature and Gene based counting of edited site in the genomic locations such as intron, exon, CDS, 3′ UTR, 5′ UTR, or unknown sites were identified based on RefSeq annotations [39].

### Pathway/network analysis

For the pathway/network analysis, we first identified all the genes that overlapped in the three normal human lung samples that contained at least one editing site in the 3′ UTR. This resulted in a total of 336 genes. We performed gene enrichment analysis using MetaCore (http://lsresearch.thomsonreuters.com/pages/solutions/1/metacore) and CytoScape BinGo (http://www.psb.ugent.be/cbd/papers/BiNGO/Home.html).

### Validation of RNA editing using Sanger sequencing

DNA and RNA were isolated from normal human lungs as described previously. Total RNA was reverse transcribed to yield cDNA using iScript RT (Biorad) and random primers as per the manufacturer's instructions (Biorad). PCR was carried out using primers that were designed flanking the editing sites to amplify at least a 400 bp product. Primers were designed such that they could be used on both genomic DNA (gDNA), and spliced cDNA. We also tagged primers with M13 primers at the end so that it could be amenable to Sanger sequencing using M13 primers alone. Amplified PCR products were purified using PCR purification kit (Qiagen) and subjected to Sanger sequencing using M13 primers at both ends using standard protocol at Moffitt Genomics facility. The potential edited sites were considered validated if the cDNA sequence at the edited site contained two peaks, (both a reference peak and an edited peak) but the corresponding gDNA contained only one reference peak. Sites were considered to be un-validated if it contained only a single reference peak in both cDNA and gDNA traces.

### Identification of editing sites in the microRNA target site

A list of potential human miRNA target sites were derived from Ensembl (GRCh37) transcripts using miRanda [40]. miRNA targets were filtered using energy < −20 and score > 155 thresholds. Using Ensembl BioMart (GRCh37), each transcript with only one validated 3′ UTR start site had its position reported. For each of those transcripts, the BioMart-reported 3′ UTR start position was added to the miRanda-reported RNA start and end coordinates to obtain the miRNA target locations. The generated miRNA target locations were parsed to determine if the previously identified RNA edited sites existed within those boundaries.

## DATA DEPOSITION

The RNA-seq data and DNA-seq data are deposited within Short Read Archive (SRA) under accession number SRP061159. This is the site where the FASTQ files will be made publically available.

## SUPPLEMENTARY MATERIAL FIGURE AND TABLES






