# Supplementary material for: Detection of canonical A-to-G editing events at 3′ UTRs and microRNA target sites in human lungs using next-generation sequencing
Source: Oncotarget. 2015 Oct 15;6(34):35726–36. doi: 10.18632/oncotarget.6132 (PMC4742137; doi:10.18632/oncotarget.6132)
Supplement: Supplementary file 1 [file oncotarget-06-35726-s001.pdf]

Detection of canonical A-to-G editing events at 3′ UTRs and microRNA target sites in human lungs using next-generation sequencing

Supplementary Material

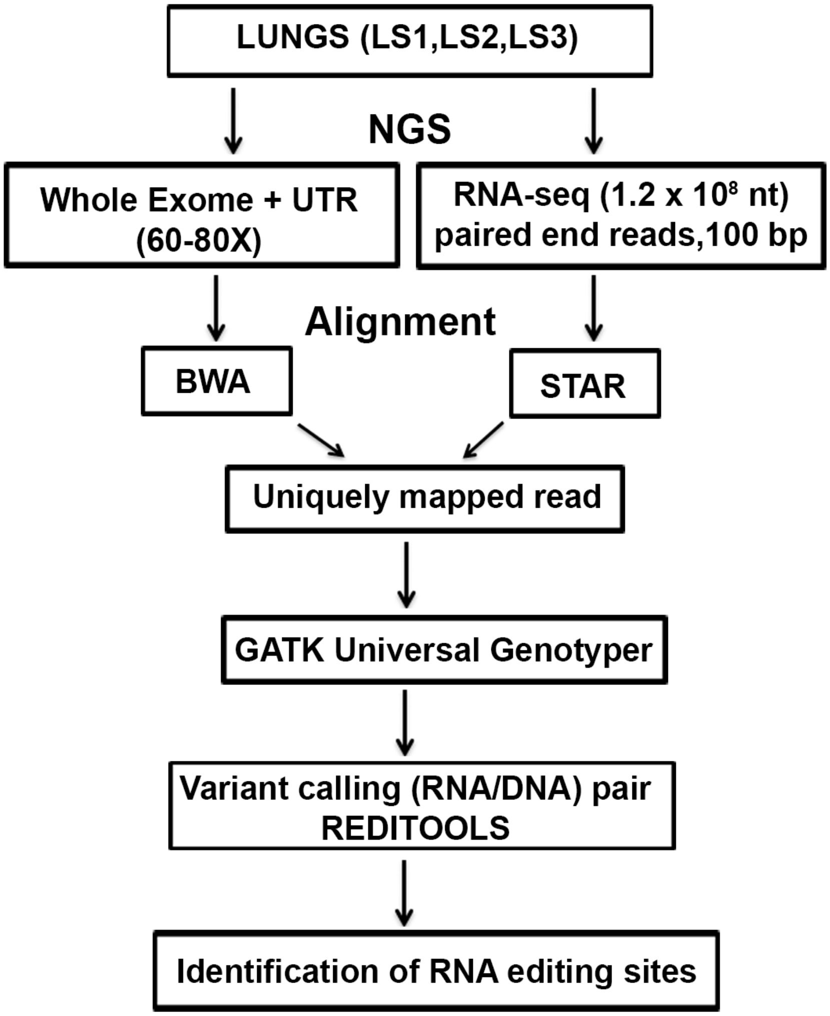

Figure S1. Bioinformatics analysis pipeline that was used for identification of editing sites. We used both (exome) DNA- and RNA-sequencing data. Both datasets were subject to a threshold of a minimum of 20X coverage. Additionally, RNA-seq data was subject to an additional threshold of a 20% alternate allele frequency, and previously identified SNPs were filtered to identify the number and distribution of RNA editing types in the three normal human lung samples.

Table S1 Number of RNA editing sites overlapping in genes of all three normal human lung samples (attached as an excel file).

Table S2 Pathway map enrichment of target genes with editing in 3' UTR. The total number of genes enriched by pathway, p-value and FDR is indicated.

Enrichment by Pathway Maps	Total Gene Number	p-value	FDR
Apoptosis and survival TNFR1 signaling pathway	6	1.26E-05	3.60E-03
Apoptosis and survival FAS signaling cascades	6	1.44E-05	3.60E-03
Translation regulation by Alpha-1 adrenergic receptors	6	4.28E-05	6.18E-03
Apoptosis and survival caspase cascade	5	5.32E-05	6.18E-03
Cytoskeleton remodeling, TGF, and WNT	8	6.18E-05	6.18E-03
Cytoskeleton remodeling	7	2.45E-04	2.04E-02
Muscle contraction GPCRs in the regulation of smooth muscle tone	6	5.22E-04	2.96E-02
Apoptosis and survival endoplasmic reticulum stress response pathway	5	5.42E-04	2.96E-02
Apoptosis and survival Granzyme B signalling	4	5.92E-04	2.96E-02
Signal transduction ERK1/2 signaling pathway	4	5.92E-04	2.96E-02

Table S3 Gene Ontology (GO) analysis of target genes with editing in the 3' UTR.

The total number of genes that mapped to the processes, p-value and FDR are shown.

Processes	Total Gene Number	p-value	FDR
Metabolic process	251	9.84E-12	4.18E-08
Macromolecule metabolic process	196	3.59E-11	7.63E-08
Cellular metabolic process	223	1.44E-10	1.63E-07
Organic substance metabolic process	231	1.92E-10	1.63E-07
Response to stress	118	2.67E-10	1.63E-07
Primary metabolic process	226	2.80E-10	1.63E-07
Cellular protein metabolic process	107	2.81E-10	1.63E-07
Protein metabolic process	125	3.06E-10	1.63E-07
Celular macromolecule metabolic process	177	5.12E-10	2.42E-07
Regulation of protein metabolic process	84	7.75E-10	3.01E-07

Table S4 miRNA binding sites that are A-to-G edited in target genes (attached as an excel file).
